# Sildenafil for congenital heart diseases induced pulmonary hypertension, a meta-analysis of randomized controlled trials

**DOI:** 10.1186/s12887-023-04180-1

**Published:** 2023-07-20

**Authors:** Ahmed K. Awad, Eman Reda Gad, Mahmoud Shaban Abdelgalil, Ahmed Saad Elsaeidy, Omar Ahmed, Merihan A. Elbadawy

**Affiliations:** 1 Faculty of Medicine, Ain-Shams University, Cairo, Egypt; 2 Faculty of Medicine, Cairo University, Cairo, Egypt; 3 Faculty of Medicine, Ain-Shams University, Cairo, Egypt; 4 Faculty of Medicine, Benha University, Benha, Egypt; 5 Sheffield Teaching Hospitals, NHS Foundation Trust, Sheffield, UK

**Keywords:** Sildenafil, Congenital heart diseases, Pulmonary hypertension, Ventricular septal defect

## Abstract

**Background:**

Sildenafil was first prescribed for angina pectoris and then for erectile dysfunction from its effects on vascular smooth muscle relaxation and vasodilatation. Recently, sildenafil has been proposed for congenital heart diseases induced pulmonary hypertension, which constitutes a huge burden on children's health and can attribute to fatal complications due to presence of unoxygenated blood in the systemic circulation. Therefore, our meta-analysis aims to further investigate the safety and efficacy of sildenafil on children population.

**Methods:**

We searched the following electronic databases: PubMed, Cochrane CENTRAL, WOS, Embase, and Scopus from inception to April 20th, 2022. Randomized controlled trials that assess the efficacy of using sildenafil in comparison to a placebo or any other vasodilator drug were eligible for inclusion. The inverse variance method was used to pool study effect estimates using the random effect model. Effect sizes are provided in the form of mean difference (MD) with 95% confidence intervals (CI).

**Results:**

Our study included 14 studies with (*n* = 849 children) with a mean age of 7.9 months old. Sildenafil showed a statistically significant decrease over placebo in mean and systolic pulmonary artery pressure (PAP) with MD -7.42 (95%CI [-13.13, -1.71], *P* = 0.01) and -8.02 (95%CI [-11.16, -4.88], P < 0.0001), respectively. Sildenafil revealed a decrease in mean aortic pressure and pulmonary artery/aortic pressure ratio over placebo with MD -0.34 (95%CI [-2.42, 1.73], *P* = 0.75) and MD -0.10 (95%CI [-0.11, -0.09], *P* < 0.00001), respectively. Regarding post corrective operations parameters, sildenafil had a statistically significant lower mechanical ventilation time, intensive care unit stay, and hospital stay over placebo with MD -19.43 (95%CI [-31.04, -7.81], *s* = 0.001), MD -34.85 (95%CI [-50.84, -18.87], *P* < 0.00001), and MD -41.87 (95%CI [-79.41, -4.33], P = 0.03), respectively. Nevertheless, no difference in mortality rates between sildenafil and placebo with OR 0.25 (95%CI 0.05, 1.30], *P* = 0.10) or tadalafil with OR 1 (95%CI 0.06, 17.12], *P* = 1).

**Conclusion:**

Sildenafil is a well-tolerated treatment in congenital heart diseases induced pulmonary hypertension, as it has proven its efficacy not only in lowering both PAP mean and systolic but also in reducing the ventilation time, intensive care unit and hospital stay with no difference observed regarding mortality rates.

**Supplementary Information:**

The online version contains supplementary material available at 10.1186/s12887-023-04180-1.

## Introduction

Present in a variety of organs, such as blood vessels, corpus cavernosum, liver, and kidney with more than eleven different families defined, phosphodiesterase (PDE) is an enzymes family which controls the activity of secondary messengers, such as cyclic adenosine monophosphate (cAMP) and cyclic guanosine monophosphate (cGMP) [1]. Thus, each specific family of PDE is specific in treating selective diseases upon acting on specific 2ndary messengers, such as PDEs 5, 6, and 9 which are selective for cGMP. Upon hydrolyzation of cGMP by PDE5, it results in a cascade establishing various cellular effects, including ion transport, endothelial permeability, and smooth muscle relaxation [1]. PDE5 inhibitors (PDE5-Is) manifest their effects by inhibiting the PDE5-dependent cGMP hydrolysis, thus increasing cGMP intracellularly. As cGMP is the main secondary messenger for both nitric oxide and natriuretic peptide systems, thus upon increasing, it results in vascular smooth muscles relaxation and vasodilatation [2].

Therefore, PDE 5 inhibitors, such as sildenafil, were first prescribed for angina pectoris and then further proven their efficacy in the treatment of erectile dysfunction (ED). As both cardiovascular diseases and ED share the same risk factors, such as age, smoking, hypertension, depression, and metabolic syndrome, and the underlying pathophysiology from endothelial dysfunction, inflammation of small blood vessels, and formation of atherosclerotic plaques. Recently, several studies have elaborated on their cardioprotective efficacy and proposed their usage in pulmonary arterial hypertension (PAH) [3, 4]. PAH can result from several congenital heart diseases (CHD), such as septal defects: ASD, VSD, or PDA which results in the right to left shunt. Without being treated, PAH can cause several fatal complications due to the unoxygenated blood presents in the systemic circulation [5]. Whether to use PDE5-Is in congenital heart diseases induced pulmonary hypertension has been a controversy emerging from its ingenious potential side effects on children [5]. Thus, our meta-analysis aimed to further investigate the safety and efficacy of sildenafil in congenital heart diseases induced pulmonary hypertension (CHD-PAH).

## Methods

This review was performed according to Preferred Reporting Items for Systematic Reviews and Meta-Analysis (PRISMA) guidelines [6], and the Cochrane handbook for systematic reviews and meta-analysis [7] and has been registered with https://doi.org/10.27405/OSF.IO/TQBXA.

### Search strategy

We conducted our search of the following databases: PubMed, Cochrane CENTRAL, WOS, Embase, and Scopus using MeSH terms and keywords for sildenafil, pulmonary hypertension, and congenital heart diseases to April 20, 2022. The full details of the systematic search strategy are illustrated in the supplementary table (1).

### Eligibility criteria

Randomized controlled trials (RCTs) assessing the efficacy of sildenafil versus placebo or other vasodilator drugs in PAH due to congenital heart diseases especially VSD that were published in peer-reviewed international journals and had enough data for qualitative and quantitative analysis were included with no language restrictions. We excluded articles published in non-peer-reviewed journals, letters to editors, observational studies, and articles that didn’t match our eligibility criteria. Our PICO was P; children aged less than 18 years old, I; sildenafil in treating pulmonary hypertension caused by congenital heart diseases, C; placebo or any other vasodilating agent, O; mean pulmonary arterial pressure (mPAP), Systolic pulmonary arterial pressure (sPAP), mean aortic pressure (mAOP), pulmonary artery and aortic ratio (PA/AO), mechanical ventilation in hours, intensive care unit (ICU) stays in hours, hospital stays in hours, and mortality.

### Data extraction

Two independent authors M.S and A.E extracted the following data about baseline characteristics from the included studies: first author name, year of publication, sample size, characteristics of participants (sex, age, and weight), type of congenital anomaly, VSD type, the dose of sildenafil, and mean pulmonary arterial pressure (mPAP). The same authors independently extracted data for the quantitative analysis; mPAP, systolic pulmonary arterial pressure (sPAP), PA/AO ratio, mechanical ventilation duration (in hours), ICU stay (in hours), hospital stay (in hours), and mortality rate.

## Risk of bias assessment

We used the Cochrane risk of bias (ROB-2) assessment tool which is described in chapter 8.5 of the Cochrane handbook [6]. This tool can detect five types of bias including selection bias, performance bias, detection bias, attrition bias, and reporting bias. We classified included articles in each domain as low, some concerns, or high bias.

### Data Analysis

Continuous and dichotomous data were extracted and pooled as mean difference (MD) and odds ratio (OR) with a 95% confidence interval (CI). The inverse-variance (IV) method was used to pool stud estimates using a random effect model. We assessed heterogeneity by I^2^ statistics. According to the Cochrane handbook, I^2^ more than 50% may represent substantial heterogeneity, in which case sensitivity analysis was performed by eliminating each study one at a time to assess the influence of each of the included studies on effect estimate. Funnel plots were used for visual investigation of publication bias. All analyses were carried out using RevMan version 5.3 [8] for windows.

## Results

### Search results

Our search strategy resulted in a total number of 4326 studies. After the title and abstract screening and removing the duplicates, 3035 articles were eliminated, and 39 full-text articles were evaluated for eligibility. Following the full-text screening, 14 [9–22] papers met our criteria and were included in our systematic review and meta-analysis (Supplementary Fig. 1).

### Risk of bias assessment

According to the ROB-2 tool, our included studies in terms of the randomization process, deviation from the intended interventions, missing outcome data, measurement of the outcome, and selection of the reported results showed five studies of low risk of bias, five of some concerns due to deviation from intended intervention or selection of reported results, and four of high risk due to deviation from intended intervention or measurement of outcomes. Further details are shown in (Supplementary Fig. 2).

### Summary of the included studies

With a mean age of 7.9 months old, our study included 849 children. All our studies were randomized controlled trials with mPAP of 60 mmHg. Ventricular septal defect (VSD) was the congenital anomaly found in all our includes studies, and associated in some studies with other anomalies such as atrial septal defect (ASD), patent ductus arteriosus (PDA), and patent foramen ovale (PFO); furthermore, more baseline characteristics are illustrated in (Table 1).

**Table 1:** Summary and baseline characteristics of included studies

Author, Year	Sample size (number)	VSD type (number)	Other congenital anomalies name (number)	Sildenafil dose	Study Design	Age, months, Mean (SD)		Weight, kg, Mean (SD)		mPAP, mmHg, Mean (SD)	
						**Sildenafil**	**comparator**	**Sildenafil**	**comparator**	**Sildenafil**	**comparator**
Namachivayam 2006 [15]	29	NA (29)	NA	0.3 to 0.5 mg/kg	RCT	67.68 (9.6)	40.32 (6)	4.6 (5.9)	4.0 (6.6)	35.1 (13.3)	31.0 (9.1)
Peiravian 2007 [17]	42	42	PDA (21) ASD (3) SAW (4) APW (2)	0.3 mg/kg	RCT	63 (56.4)	47.64 (38.4)	14.33 (6.86)	12.88 (5.40)	76.8 (17.4)	81.7 (16.3)
Fraisse 2011 [13]	17	NA (4)	ASD (1) Complete AVC (7) Truncus arteriosus (1) Down syndrome (7) Others (4)	10 mg/ml	RCT	5 months (range 3–168 months)		5.2 kg (range 3.9–60 kg)		33 (10)	37 (10)
El Midany 2013 [11]	101	perimembranous (51) subarterial (24) double committed (3) muscular (23)	PDA (32) ASD (19) main pulmonary artery aneurysm (1)	0.5 mg/kg to 2 mg/kg	RCT	116.04 (44.4)	147.96 (71.16)	6.5 (1.4)	7.3 (2)	75.4 (7.8)	74.6 (8.2)
Farah 2013	48	40	AVSD (6) APW (2)	0.3 mg/kg	RCT	162 (154.8)	147.6 (139.2)	7.1 (3.1)	6.2 (2.3)	74 (15)	78 (14)
Barst, 2011	234	NA (234)	Congenital systemic to pulmonary shunt with SaO2 ≥ 88% at rest (85)	≤ 20 kg, 10 mg > 20 kg, 20 mg	RCT	NA	NA	NA	NA	63 (22)	59 (22)
Kuntartiwi 2015 [14]	36	NA (14)	ASD (8) PDA (12)	≤ 8 kg, 5 mg 8 to 20 kg, 10 mg > 20 to 45 kg, 20 mg ≥ 45 kg,40 mg	RCT	48 (300)	72 (384)	11.5 (20)	13 (20)	59 (12.1)	53.5 (13.7)
Sharma 2015	46	NA (46)	NA	0.5 mg/kg	RCT	456 (36)	504 (40.8)	12.6 (7.1)	12.8 (7.6)	44.2 (7.2)	46.2 (8.4)
Bigdelian 2017 [10]	63	Per membranous (60) Muscular (3)	VSD + ASD (6) VSD + PDA (47)	0.3 mg/kg	RCT	64.8 (6)	64.8 (7.2)	6.6 (0.6)	6.4 (0.6)	71.3 (2.9)	75.(3.0)
Sabri 2017 [18]	42	Large VSD (41) Tow muscular VSDs (1)	ASD (13) PDA(19) PFO (1)	1–3 mg/kg/day	RCT	7.38 (4.38)	7.33 (4.56)	5.90 (1.17)	5.86 (1.59)	60.38 (14.94)	60.52 (13.51)
Patel 2020 [16]	30	Perimembrano-us (PM) (15) subaortic (7) muscular (5) inlet VSD (2)	left superior vena cava (1) Down's syndrome (4)	0.5 mg/kg	RCT	144 (37.2)	144 (67.2)	6 (2.1)	6.8 (3.3)	69.83 (6.38)	68.81 (5.31)
Palli 2014	77	NA (77)	Congenital shunts (14 simple, 35 mixed, and 28 complex)	1–2 mg/kg/day	RCT	19.9 (5.3)	21.7 (7.8)	NA	NA	NA	NA
Vassalos 2011 [20]	24	NA (13)	AVSD (11) Trisomy 21 (12)	0.5 mg.kg	RCT	7.44 (6.84)	5.04 (2.04)	NA	NA	NA	NA
Bhasin 2017 [22]	60	Large VSD (60)	NA	0.5 mg/kg	RCT	14.53 (12.62)	15.83 (13.06)	8.98 (3.75)	8.95 (4.50)	54.367 (10.005)	60.633 (10.469)

*SD* Standard deviation, *NA* Not available, *mPAP* mean Pulmonary arterial pressure, *PDA* Patent ductus arteriosus, *ASD* Atrial septal defect, AVC Atrioventricular canal, *AVSD* Atrioventricular septal defect, *SAW* Sub Aortic Web, *APW* Aortopulmonary window, *PFO* Patent foramen ovale, *RCT* Randomized clinical trial

### Meta-analysis

#### Mean pulmonary arterial pressure (mPAP)

**Fig. 1:**
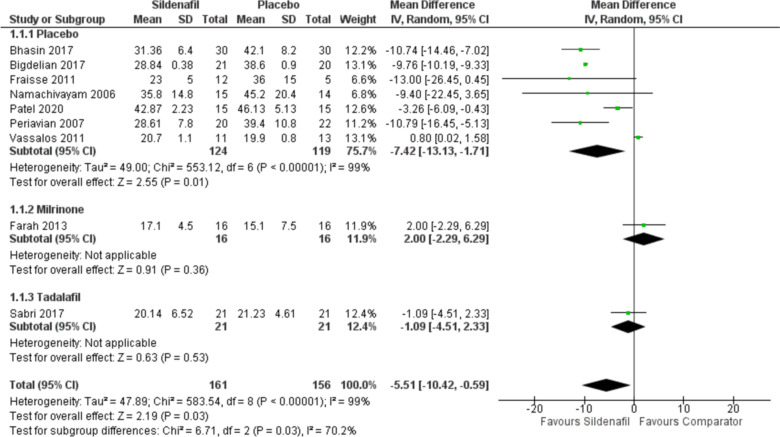
Forest plot of the analysis of mPAP

Our analysis of mPAP includes nine studies with 161 participants in the sildenafil group and 156 in the comparator group showing a statistically significant decrease in mPAP for sildenafil over placebo with MD -7.42 (95%CI [-13.13, -1.71], *P* = 0.01, I^2^ = 99%). Subgrouping comparing sildenafil with milrinone and tadalafil showed a statistically insignificant increase with sildenafil when compared with milrinone with MD 2 (95%CI [-2.29, 6.29], *P* = 0.36), yet decrease when compared to tadalafil MD -1.09 (95%CI [-4.51, 2.33], *P* = 0.53) (Fig. 1).

#### Systolic pulmonary arterial pressure (sPAP)

**Fig. 2:**
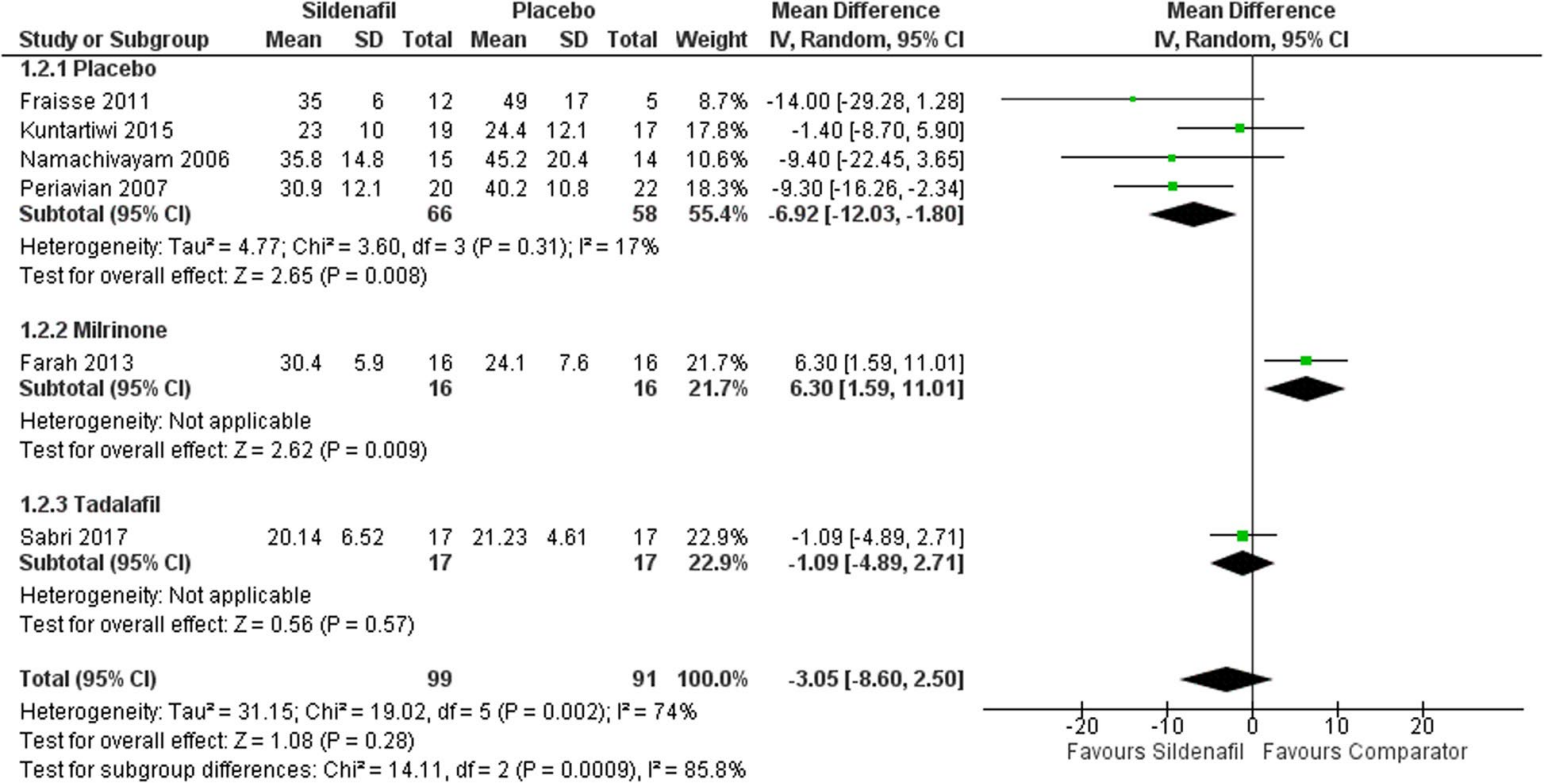
Forest plot of the analysis of sPAP

Our analysis of sPAP includes six studies with 99 participants in the sildenafil group and 91 in the comparator group showing a statistically significant decrease in sPAP for sildenafil over placebo with MD -6.92 (95%CI [-12.03, -1.80], P = 0.008, I^2^ = 17%). Subgrouping comparing sildenafil with milrinone and tadalafil showed a statistically significant increase with sildenafil when compared with milrinone with MD 6.30 (95%CI [1.59, 11.01], P = 0.009), yet insignificant decrease when compared to tadalafil MD -1.09 (95%CI [-4.89, 2.71], *P* = 0.57) (Fig. 2).

#### Mean Aortic pressure (mAOP)

**Fig. 3:**
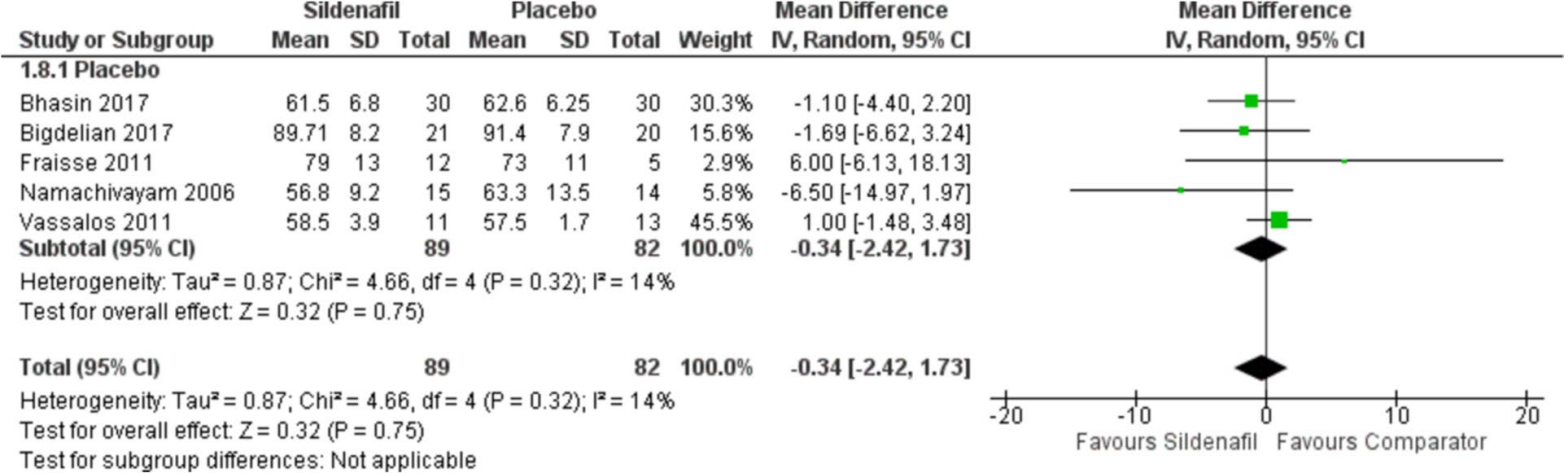
Forest plot of the analysis of mAOP

Our analysis of mAOP includes five studies with 89 participants in the sildenafil group and 82 in the comparator group showing a decrease in mAOP for sildenafil over placebo with MD -0.34 (95%CI [-2.42, 1.73], *P* = 0.75, I^2^ = 14%), yet without significance (Fig. 3)**.**

#### Pulmonary artery and aortic ratio (PA/AO)

**Fig. 4:**
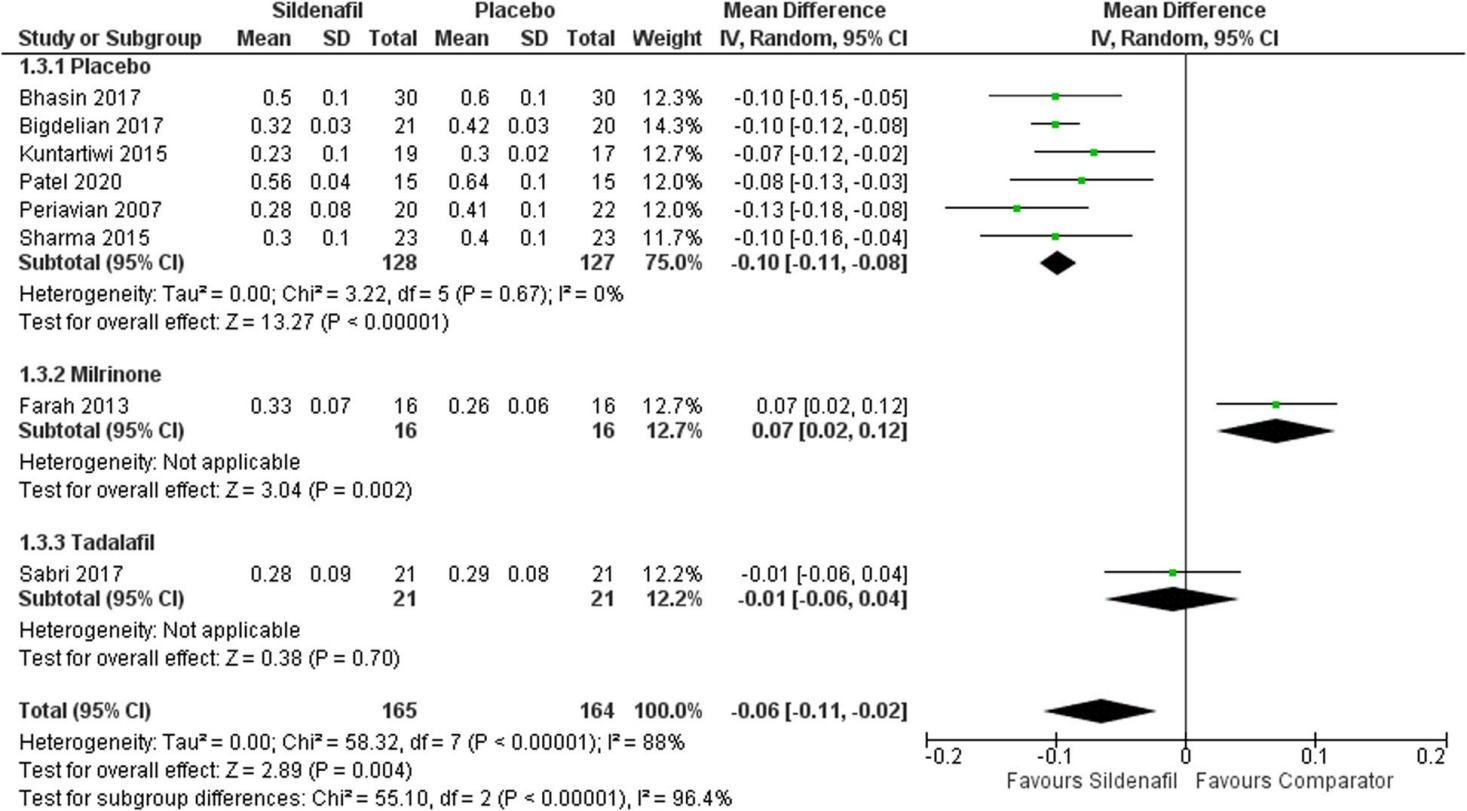
Forest plot of the analysis of (PA/AO)

Our analysis of (PA/AO) includes eight studies with 165 participants in the sildenafil group and 164 in the comparator group showing a statistically significant decrease in PA/OA favoring sildenafil over placebo with MD -0.10 (95%CI [-0.11, -0.08], *P* < 0.00001, I^2^ = 0). Furthermore, subgrouping sildenafil versus milrinone and tadalafil showed a statistically significant increase for sildenafil with MD 0.07 (95%CI 0.02–0.12, *p* = 0.002) and an insignificant decrease for sildenafil with MD -0.01 (95%CI -0.06–0.04, *p* = 0.70), respectively (Fig. 4).

#### Mechanical ventilation during hours

**Fig. 5:**
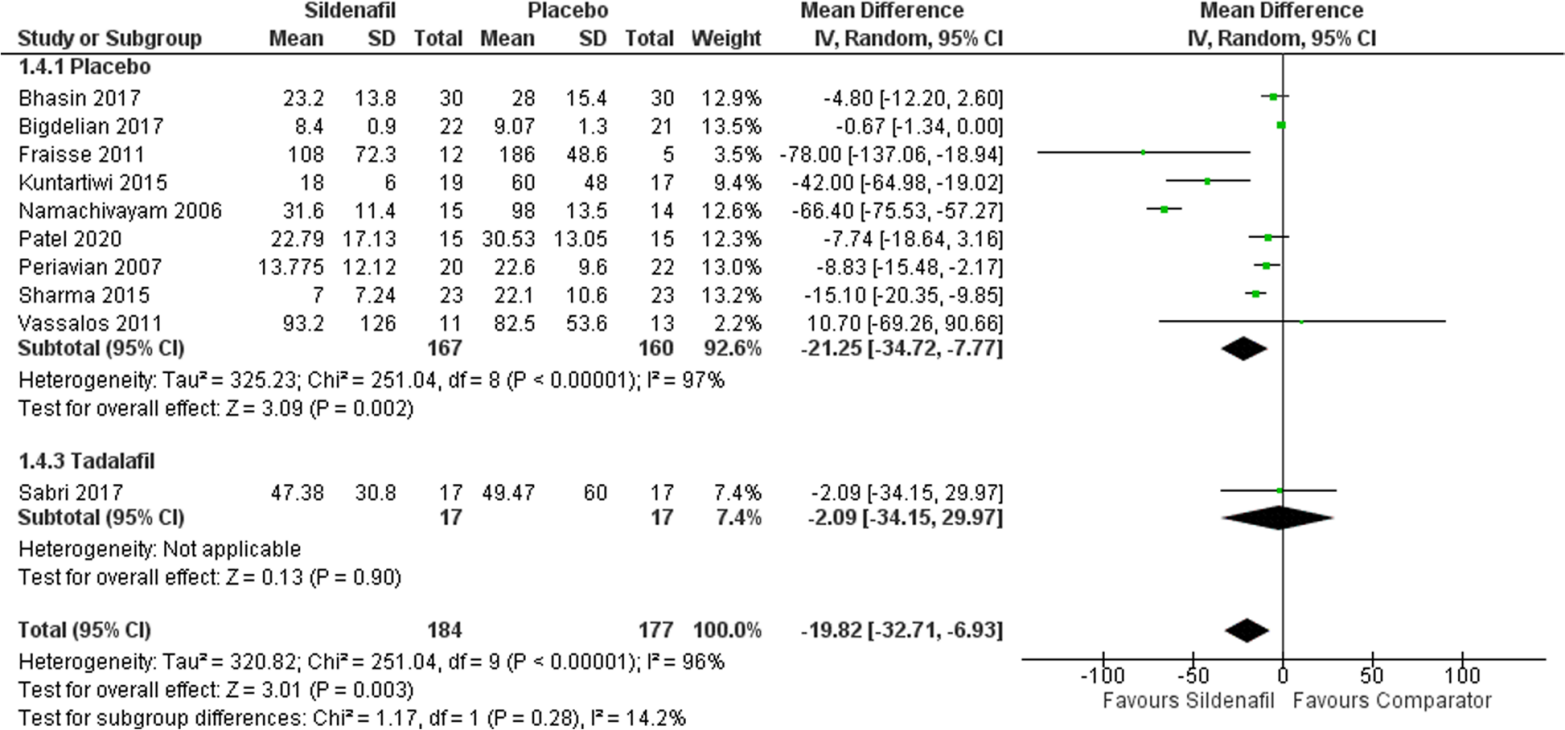
Forest plot of the analysis of mechanical ventilation

Our analysis of mechanical ventilation in hours includes ten studies with 184 participants in the sildenafil group and 177 in the comparator group showing a statistically significant lower mechanical ventilation time for sildenafil over placebo with MD -21.25 (95%CI [-34.72, -7.77], P = 0.002, I^2^ = 97%). However, comparing sildenafil with tadalafil showed an insignificant decrease favoring sildenafil with MD -2.09 (95%CI [-34.15, 29.97], P = 0.90) (Fig. 5). Moreover, mechanical ventilation showed an asymmetrical distribution of the studies toward the positive reporting results (supplementary Figs. 3).

#### ICU stays in hours

**Fig. 6:**
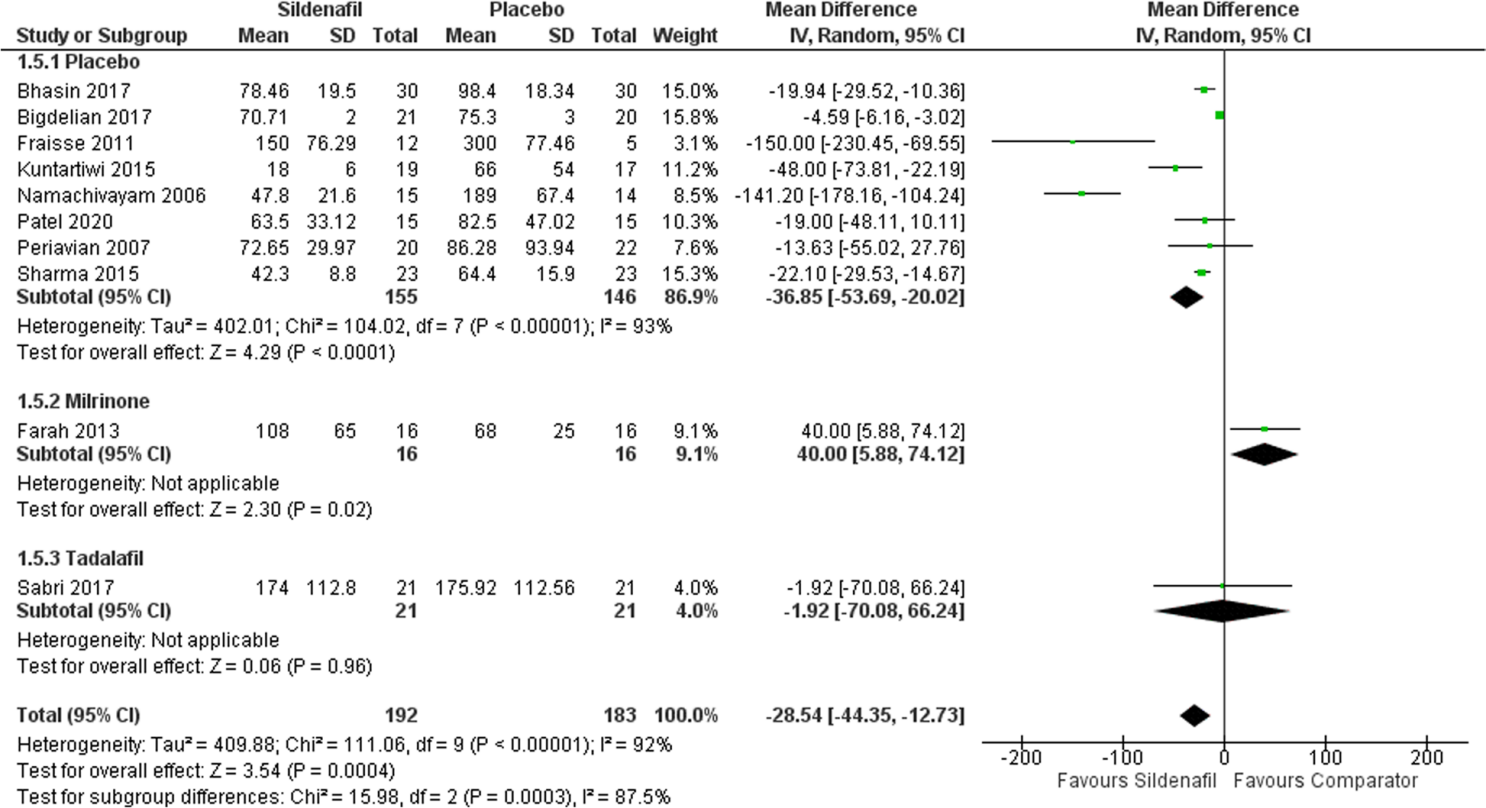
Forest plot of the analysis of ICU stays

Our analysis of ICU stay-in hours includes ten studies with 192 participants in the sildenafil group and 183 in the comparator group showing a statistically significant decrease in ICU stay for sildenafil over placebo with MD -36.85 (95%CI [-53.69, -20.02], P < 0.00001, I^2^ = 93%). Furthermore, when compared to milrinone, sildenafil showed significantly higher ICU stay time with MD 40 (95%CI [5.88, 74.12], P = 0.02), yet an insignificant decrease when compared to tadalafil with MD -1.92 (95%CI [-70.08, 66.24], p = 0.96) (Fig. 6)**.** Moreover, ICU stay analysis showed a symmetrical distribution of the studies according to our funnel plot (supplementary Figs. 4).

#### Hospital stays in hours

**Fig. 7:**
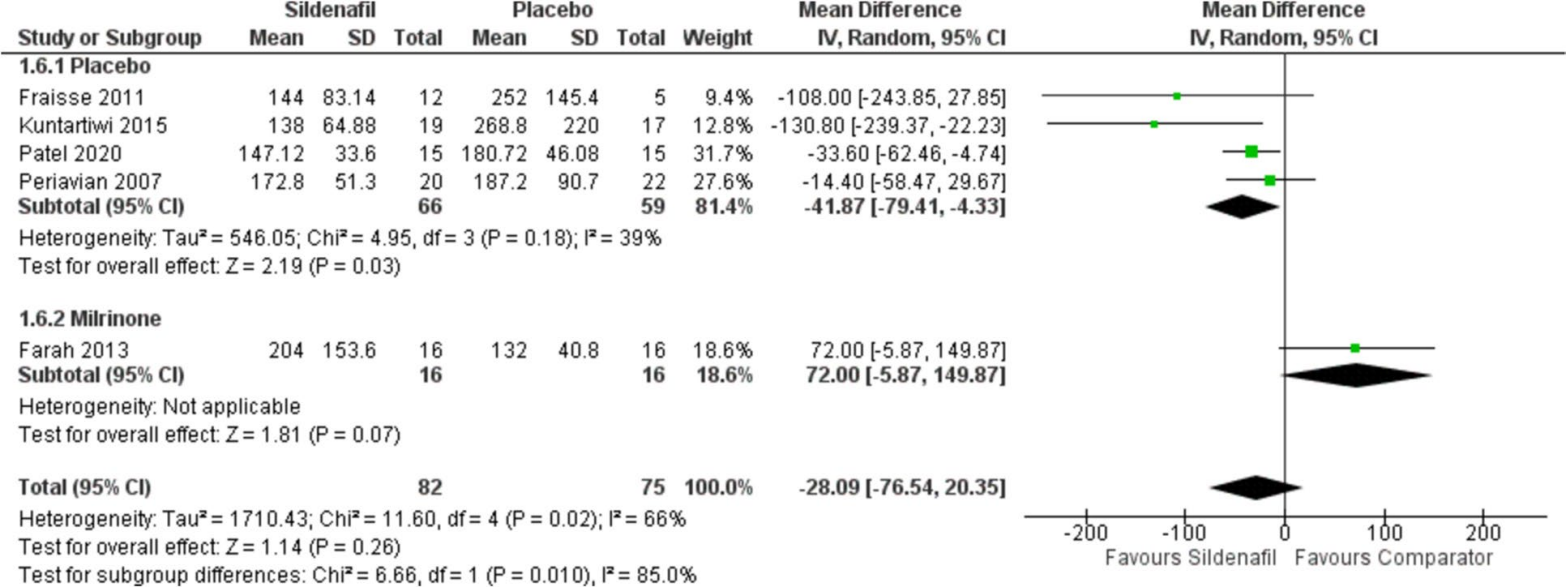
Forest plot of the analysis of hospital stays

Our analysis of hospital stays in hours includes five studies with 82 participants in the sildenafil group and 75 in the comparator group showing a statistically significant decrease in hospital stay for sildenafil over placebo with MD -41.87 (95%CI [-79.41, -4.33], P = 0.03, I^2^ = 39%). However, when compared to milrinone, sildenafil showed insignificant higher hospital stay rates with MD 72 (95%CI [-5.87, 149.87], *P* = 0.07) (Fig. 7)**.**

#### Mortality

**Fig. 8:**
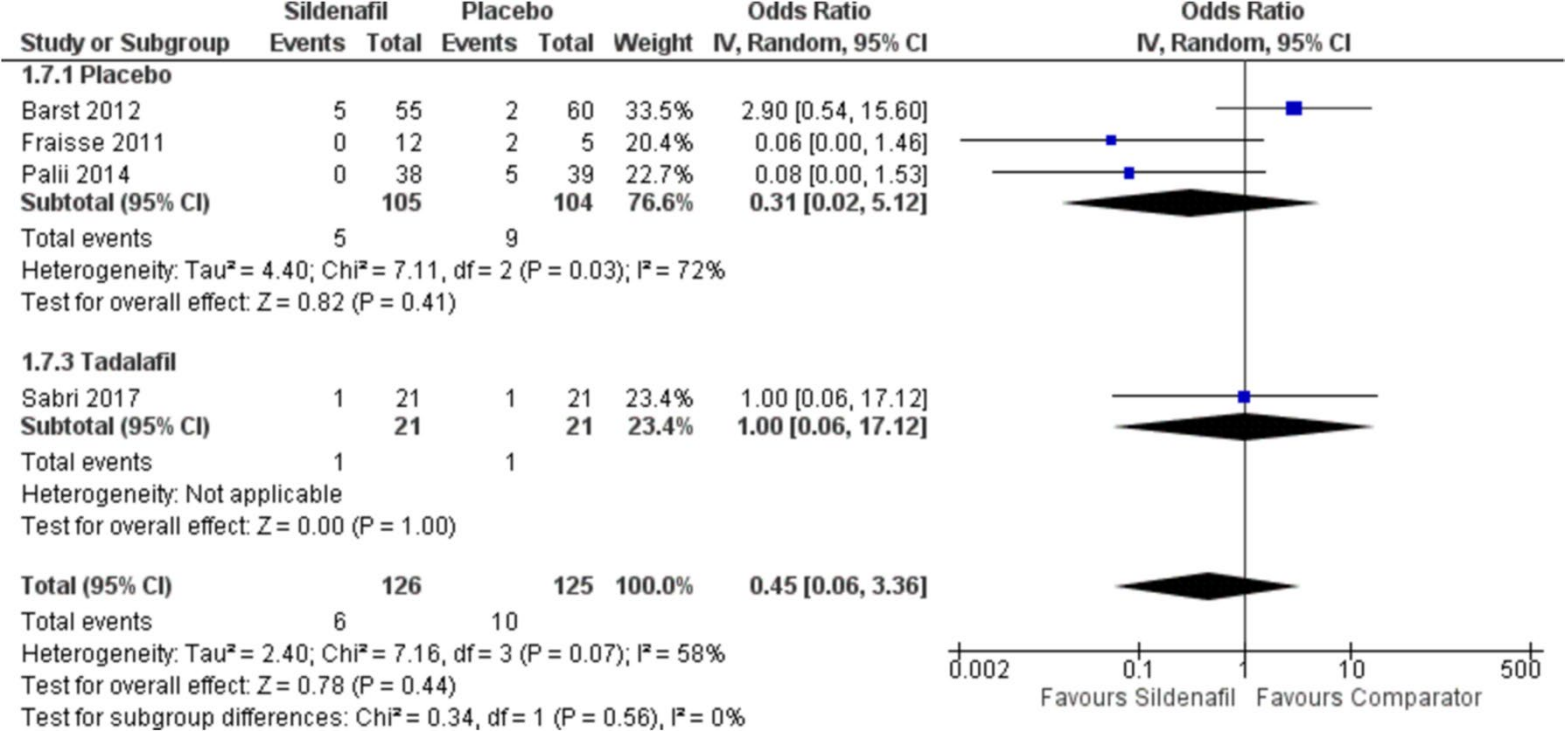
Forest plot of the analysis of mortality rates

Our analysis of mortality rates includes four studies with 126 participants in the sildenafil group and 125 in the comparator group showing no difference in mortality rates between sildenafil and placebo with OR 0.31 (95%CI 0.02, 5.12], *P* = 0.41, I^2^ = 72%). Moreover, sildenafil showed no difference between tadalafil in risk of mortality with OR 1 (95%CI 0.06, 17.12], *P* = 1) (Fig. 8).

## Discussion

Our manuscript is the most comprehensive meta-analysis done today including 14 studies, with a total of 849 children, comparing the efficacy of using sildenafil in pulmonary hypertension associated with congenital heart diseases. Our meta-analysis showed a significant decrease favoring the sildenafil group in mPAP, sPAP, PO/AO, mechanical ventilation duration, ICU stays, and hospital stays, yet an insignificant decrease in mAOP and rates of mortality. When compared to milrinone, sildenafil showed higher mPAP, PO/AO, sPAP, ICU stay, and hospital stay; nevertheless, when compared to tadalafil, sildenafil showed lower mPAP, sPAP, PO/AO, mechanical ventilation time, and ICU stay with no difference in mortality rates.

Furthermore, studies that have not been included in our analysis yet in our systematic review including Brast et al. whom conducted STARTS-1 [9] and 2 [23] randomized controlled trials. In STARTS-1, they compare the effect of sildenafil in several doses: low, medium, and high orally 3 times daily for 16 weeks versus placebo on patients with pulmonary arterial hypertension (PAH). Congenital anomaly-induced hypertension was found in 39 out of 60 patients in the placebo group and 117 out of 174 patients in the sildenafil group. Overall, sildenafil was well tolerated for pediatric PAH with better exercise capacity and hemodynamics with medium and high dose sildenafil with overall profile favoring medium dose sildenafil. In STARTS-2 [23], a 3-year follow-up showed that all sildenafil dose groups exhibited satisfactory survival for children with PAH, although higher unexplained mortality rates were observed with higher compared with lower sildenafil doses.

Moreover, El-Midany et al. conducted a study enrolling 101 patients randomly assigned to either receiving sildenafil pre- and post-operative correction of a congenital cardiac defect causing PAH (*n* = 51) versus post-operative only (*n* = 50). Although a slight difference was observed favoring the pre-post-operative group in terms of mPAP, they found no statistical difference between the two groups in terms of mechanical ventilation time, ICU, and hospital stay time.

Pulmonary arterial hypertension (PAH) is a progressive pathology that can cause various complications without treatment as a right ventricular failure and even death [24]. There are several etiologies for PH, they can be classified into five groups: (1) PAH secondary to congenital heart disease (PAH-CHD); (2) illnesses that emerge from either lung diseases or hypoxia (3) a reaction to left-sided heart disease; (4) a grouping for unclear, unknown, multifactorial mechanisms and (5) chronic thromboembolism [25]. CHD leads to increased blood flow to the pulmonary circulation that results in vascular dilation, endothelial damage, shear stress, impairment in the production of vasodilators such as NO endothelin-1 and prostacyclin, and activation of remodeling pathway that results in smooth muscle hypertrophy and fibrosis due to activation of fibroblasts [26]. Early changes to the vascular bed can be reversible if the underlying congenital heart defect is repaired within months, and the pulmonary vascular resistance (PVR) can return to normal values within 1 year [27].

PAH-CHD results from congenital septal defects such as ASD, VSD, or PDA which is called right to left shunt with the most common cause being ASD [28, 29]. In right to left shunt, there is increased blood flow to pulmonary circulation leading in last to pulmonary vasoconstriction and increased right atrial/ventricular pressure. If systolic pressure between both ventricles is equal, this is Eisenmenger's Syndrome. Reversal of the shunt in Eisenmenger's Syndrome relieves the pressure in the right ventricle but results in cyanosis due to unoxygenated blood entering the systemic circulation and other life-threatening complications. Medical or surgical interventions are to be done before Eisenmenger's Syndrome development where there is established PAH and surgical correction in the meantime can aggravate the condition to "the point of no return” [27, 28].

Zhang et al. [30], in their meta-analysis of 15 studies involved, assessed the efficacy of sildenafil in children with pulmonary arterial hypertension (PAH) caused by either idiopathic, congenital heart diseases, persistent pulmonary hypertension of the newborn, or high altitude heart disease, and they showed that mPAP and length of hospital stay were not statistically different between sildenafil and control groups. On the other hand, our study shows statistically different mPAP and length of hospital stay for the sildenafil group. Moreover, the pooled estimate favored that sildenafil reduced the duration of mechanical ventilation time and length of ICU stay and these findings are consistent with ours. Chinwe Unegbu et al. [31], in their meta-analysis, which included 8 RCTs, assessed the effect of PDE5 inhibitors on pulmonary hypertension. Four of the included studies involved patients with CHD-associated PH. 3 studies reported no significant difference in hospital stay, unlike our study. They reported improvement in mPAP by catheterization measurement between both groups which are consistent with our finding.

Several other phosphodiesterases (PDE) inhibitors have been introduced as a solution to PAH, such as milrinone and tadalafil. Milrinone is a type III PDE-3 inhibitor with vasodilating and inotropic effects; first introduced as a treatment for end-stage heart failure as infusion therapy or bridge to cardiac transplant [32]. Due to its vasodilating effects, it has been proposed for neonates' pulmonary hypertension showing promising results as a comparator for sildenafil. Milrinone has been associated with lower mPAP, ICU stays, and hospital stay [12] which is consistent with our findings, yet higher sPAP, and mAOP when compared to sildenafil. Furthermore, A study conducted by El-Ghandour et al. [33] comparing sildenafil to milrinone in neonates with persistent PAH showed better mPAP, sPAP, and right ventricular function with milrinone over sildenafil, with the best results being associated with dual sildenafil and milrinone therapy.

Tadalafil is a type V PDE-5 inhibitor with vasodilation effects primarily introduced for erectile dysfunction and benign prostatic hyperplasia [34], then proposed for treating PAH caused by congenital heart anomalies [18]. However, it showed no significant improvement over sildenafil in any of the hemodynamics parameters. Moreover, in a recent network meta-analysis [35] conducted on PAH in adults, tadalafil was associated with a low change in 6-min walking distance, besides being associated with high clinical worsening compared to other PDE-inhibitors. Bosentan with Iloprost and Bosentan with Sildenafil was the better combinations associated with the lowest mPAP.

### Limitations

Our study has many limitations, firstly the substantial heterogeneity reported in some of our analyses which we tried to solve by sensitivity analysis, secondly baseline differences observed between included studies through slight overlap in one study between CHD-induced PAH and idiopathic PAH, different associated congenital anomalies with VSD, differences in sildenafil regimen which we solved by implementing the inverse variance method and random effect model.

## Conclusion

Sildenafil is a safe, inexpensive, and well-tolerated treatment in pulmonary hypertension induced by congenital heart diseases especially VSD, as it has proven its efficacy not only in lowering the mPAP and sPAP but also in reducing the mechanical ventilation time, ICU and hospital stay with no difference observed regarding mortality rates.

## Supplementary Information

**Additional file 1: Supplementary Figure. 1.** Prisma flow diagram.

**Additional file 2: Supplementary Figure 2.** Risk of bias diagram.

**Additional file 3: Supplementary Figure 3.** Funnel plots of the mechanical ventilation analysis.

**Additional file 4: Supplementary Figure 4.** Funnel plots of the ICU stay analysis

**Additional file 5. **

## Data Availability

All data generated or analysed during this study are included in this published article and its supplementary information files.
