# Supplementary figures and images for: Sildenafil for congenital heart diseases induced pulmonary hypertension, a meta-analysis of randomized controlled trials
Source: BMC Pediatr. 2023 Jul 20;23:372. doi: 10.1186/s12887-023-04180-1 (PMC10360284; doi:10.1186/s12887-023-04180-1)

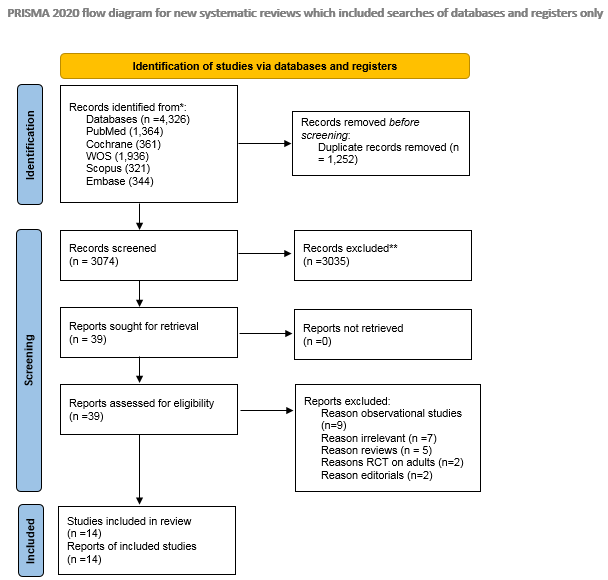

Supplement: Supplementary file 1 — Additional file 1: Supplementary Figure. 1. Prisma flow diagram. [file 12887_2023_4180_MOESM1_ESM.png]

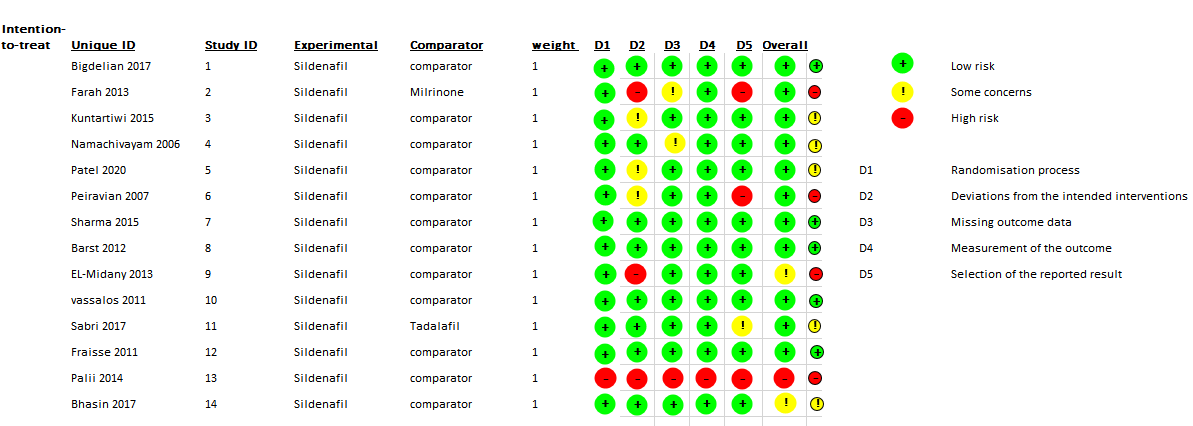

Supplement: Supplementary file 2 — Additional file 2: Supplementary Figure 2. Risk of bias diagram. [file 12887_2023_4180_MOESM2_ESM.png]

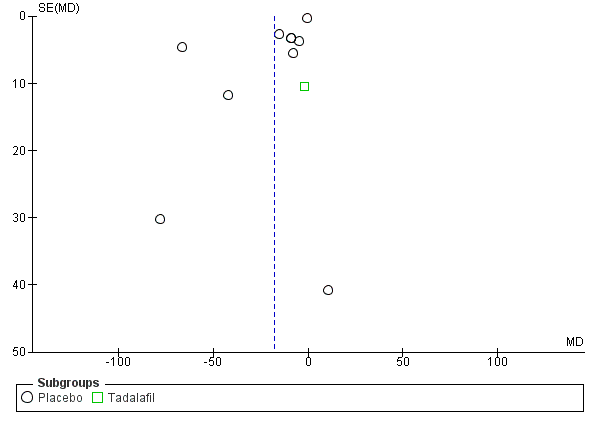

Supplement: Supplementary file 3 — Additional file 3: Supplementary Figure 3. Funnel plots of the mechanical ventilation analysis. [file 12887_2023_4180_MOESM3_ESM.png]

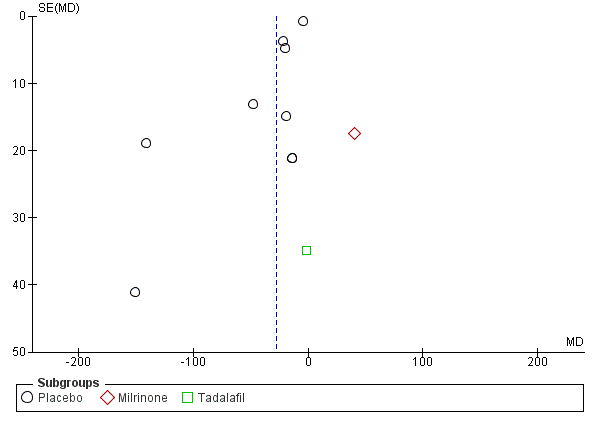

Supplement: Supplementary file 4 — Additional file 4: Supplementary Figure 4. Funnel plots of the ICU stay analysis [file 12887_2023_4180_MOESM4_ESM.png]
