# Supplementary material for: Sildenafil for congenital heart diseases induced pulmonary hypertension, a meta-analysis of randomized controlled trials
Source: BMC Pediatr. 2023 Jul 20;23:372. doi: 10.1186/s12887-023-04180-1 (PMC10360284; doi:10.1186/s12887-023-04180-1)
Supplement: Supplementary file 5 — Additional file 5. [file 12887_2023_4180_MOESM5_ESM.docx]

**Search strategy:**

1-((“sildenafil citrate” OR “Citrate, Sildenafil” OR “NCX-911” OR “NCX 911” OR “NCX911” OR “Revatio” OR “Sildenafil” OR “UK 92480 10” OR “UK 9248010” OR “UK 92,480 10” OR “Homosildenafil” OR “Hydroxyhomosildenafil” OR “Viagra” OR “Acetildenafil” OR “Sildenafil Lactate” OR “Lactate, Sildenafil” OR “Sildenafil Nitrate” OR “Nitrate, Sildenafil” OR “Desmethyl Sildenafil” OR “Sildenafil, Desmethyl” OR “Desmethylsildenafil” OR “Vizarsin” OR “Granpidam” OR “AM-sildenafil” OR “Accel-sildenafil” OR “Ag-sildenafil” OR “Apo-sildenafil”) NOT combin*)

2-(“Pulmonary hypertension” OR "Hypertension, Pulmonary"[Mesh])

3-(“Pulmonary circulation” OR “Pulmonary Blood Flow” OR “Blood Flow, Pulmonary” OR “Flow, Pulmonary Blood” OR “Pulmonary Blood Flows” OR “Respiratory Circulation” OR “Circulation, Pulmonary” OR “Circulation, Respiratory” OR "Pulmonary Circulation"[Mesh])

4-(“Hypertension” OR “Blood Pressure, High” OR “Blood Pressures, High” OR “High Blood Pressure” OR “High Blood Pressures”)

5- #4 AND #3

6- #2 OR #5

7-(“systemic-to-pulmonary shunts” OR “Septal defects” OR “Right to left shunt” OR “Reverse shunt” OR "Heart Septal Defects"[Mesh] OR "Heart Septal Defects, Ventricular"[Mesh] OR “Ventricular Septal Defect” OR “Ventricular Septal Defects” OR “Defect, Ventricular Septal” OR “Septal Defect, Ventricular” OR “Septal Defects, Ventricular” OR “Intraventricular Septal Defects” OR “Defect, Intraventricular Septal” OR “Defects, Intraventricular Septal” OR “Intraventricular Septal Defect” OR “Septal Defect, Intraventricular” OR “Septal Defects, Intraventricular”)

8-( “Eisenmenger's syndrome” OR “Complex, Eisenmenger” OR “Eisenmenger's Complex” OR “Complex, Eisenmenger's” OR “Eisenmengers Complex” OR “Eisenmenger's Syndrome” OR “Eisenmengers Syndrome” OR “Syndrome, Eisenmenger's” OR “Eisenmenger Syndrome” OR “Syndrome, Eisenmenger”)

9- #7 OR #8

10- #1 AND (#6 OR #9)

**The Number of results from each database**:

PubMed (1364)

Cochrane (361)

WOS (1936)

Scopus (321)

Embase (344)
